# Factors Influencing Physical Activity in Children and Youth with Special Health Care Needs: A Pilot Study

**DOI:** 10.1155/2012/583249

**Published:** 2012-05-07

**Authors:** Katie Feehan, Margaret E. O'Neil, Diana Abdalla, Maria Fragala-Pinkham, Monica Kondrad, Zekarias Berhane, Renee Turchi

**Affiliations:** ^1^Drexel School of Public Health, Drexel University, 245 N. 15th Street, Mail Stop 660, Philadelphia, PA 19102, USA; ^2^Drexel College of Nursing and Health Professions, Drexel University, 245 N. 15th Street, Mail Stop 1030, Philadelphia, PA 19102, USA; ^3^The Research Center, Franciscan Hospital for Children, 30 Warren Street, Brighton, MA 02135, USA; ^4^Department of Pediatrics, St. Christopher's Hospital for Children, 3601 A Street, Philadelphia, PA 19134, USA

## Abstract

*Background*. Evidence suggests that children and youth with special health care needs (CYSHCN) have decreased physical activity compared to peers. This study describes weight status and physical activity in CYSHCN and identifies factors associated with physical activity and community resources to promote physical activity. *Methods*. Parents (*n* = 21) and CYSHCN (*n* = 23) were recruited from a pediatric clinic. The most prevalent diagnoses were autism (*n* = 7, 30%) and cerebral palsy (*n* = 3, 13%). Interviews were conducted with parents for information on physical activity and community resources. Children's height and weight were measured to calculate body mass index (BMI). *Results*. The majority of CYSHCN (*n* = 13, 59%) were obese. CYSHCN did not meet recommended levels of 60 minutes of daily physical activity and engaged in more screen time than recommended. More children with cognitive/behavioral/emotional diagnoses were obese compared to children with physical/medical diagnoses. A majority of parents (*n* = 16, 73%) indicated their CYSHCN need more supervision to participate in physical activity in community programs. *Conclusion*. The majority of CYSHCN in this study were obese and sedentary. Resources to promote physical activity are needed for this population.

## 1. Introduction

A major emphasis in health care today is health promotion and disease prevention driven, in part, by the increased prevalence of childhood overweight and obesity and decreased physical activity levels among children [1]. Children and youth with special health care needs (CYSHCN) are at an increased risk for obesity and inactivity compared to their peers with typical development [2–4]. CYSHCN may have physical, cognitive, and/or emotional conditions that limit their abilities to be physically active, which may increase risk for overweight and obesity. CYSHCN are defined by the federal Maternal and Child Health Bureau as, *“those who have or are at increased risk for a chronic physical, developmental, behavioral, or emotional conditions and who also require health and related services of a type or amount beyond that required by children generally” *[5]. Health promotion strategies for CYSHCN may not be addressed in primary care or in rehabilitation services due to time constraints and competing chronic and/or acute medical needs. It is especially important to promote healthy weight in CYSHCN because chronic secondary conditions accompanying overweight and obesity may lead to health problems that limit independence [4].

Health consequences of obesity in childhood and adolescence include high blood pressure and high cholesterol, which are risk factors for cardiovascular disease (CVD) [6], increased risk of decreased glucose tolerance, insulin resistance and type 2 diabetes [7], breathing problems, such as sleep apnea, and asthma [8, 9] joint and musculoskeletal problems [8, 10], fatty liver disease, gallstones, and gastro-esophageal reflux [7, 8], and risk of social and psychological problems, such as discrimination and poor self-esteem [7, 11, 12]. Children who are obese have a high likelihood of being obese as adults [13–15] and may be at risk for serious health conditions such as heart disease, diabetes, and some cancers [16].

The current prevalence of obesity in children with typical development has increased from 5% to 17%, more than a threefold increase in the last 20–30 years [17]. Although overweight and obesity are known detriments to overall health, there is no national statistic of overweight or obesity specific to CYSHCN. The National Health and Nutrition Examination Survey (NHANES) database has been examined to determine overweight and obesity in children with developmental disorders and functional limitations [2]. Findings suggest that children with physical activity limitations were more than twice as likely to be overweight compared to children without these limitations. Responses from an online health promotion survey among adolescents with special health care needs indicated that 16.8% were obese and 19.3% were overweight compared to national database on typically developing peers where 13% were obese and 15.8% were overweight [18].

Physical inactivity is a known risk factor for overweight and obesity for all children [19] and studies suggest CYSHCN participate less in physical activity than their typically developing peers [20]. This is in part due to the impairments that CYSHCN experience because of their medical conditions/diagnoses and because of barriers to physical activity in the physical or built environment. Additional barriers to physical activity in the built environment include inaccessible playgrounds (nonadaptive equipment) and inaccessible school and work environments [20, 21]. Neighborhood characteristics such as crime and traffic patterns also pose barriers to outdoor physical activity as reported by parents of CYSHCN who were overweight or obese [18].

**Figure 1:**
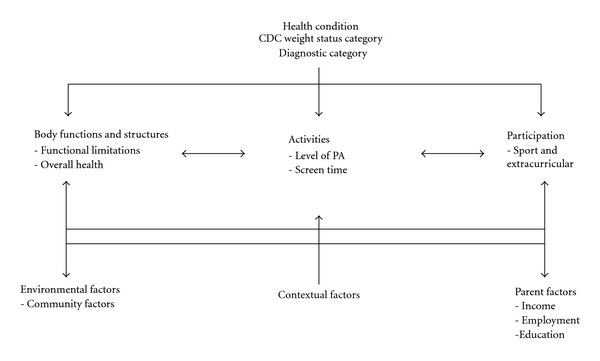
Modified ICF model.

The social and/or family environment is important to facilitate healthy behaviors in children. Parent health behaviors establish norms and set routines that can influence a child's level of physical activity [22]. Parents monitor the health behaviors of their children and are an appropriate source for information on child health behaviors [22]. Moreover, parents of CYSHCN may be more invested in their child's health-related behaviors, activities, and services due to the chronicity and intensity of their child's health condition(s) [23]. However, the social and family environment may pose barriers to physical activity and healthy lifestyles. For example, in the social environment lack of necessary staff to provide a safe and supportive environment during organized physical recreation and highly competitive team sports may pose barriers that exclude CYSHCN from participating in active leisure [20]. The family environment may present barriers to physical activity for CYSHCN if parents do not have the time or financial resources for sporting equipment or membership fees. Parents may have limited social support to be sure their children get to participate in active recreation (i.e., a single-mother may be the head of the household) or families may live in poverty. These kinds of family factors present barriers to physical activity and are associated with higher levels of obesity in CYSHCN [24–26]. In planning and implementing this pilot study, the International Classification of Functioning Model (ICF) was used as the guiding conceptual framework. The ICF Model is an enablement model that uses a holistic perspective to focus on child's *abilities* given his or her health condition(s) [27]. This model consists of personal dimensions of health (body functions and body structure; activity; participation) and the contextual factors (physical and social) that may influence personal health outcomes. As illustrated in Figure 1, the ICF model was critical to frame the study and help identify personal and environmental factors that may influence physical activity.

Families seek health and medical advice from their children's primary care providers (PCPs) [28]. They often look to the PCPs for advice and resources to promote their children's health [28]. PCPs have indicated that they need more information and training to provide effective interventions and give appropriate guidance for patients and their families [29]. Therefore, it is key to learn from parents the resources that they use and those they need to promote physical activity and health in their children. It is important to develop a community resource database to support and inform clinical practice so that PCPs can direct families to available, accessible resources to promote healthy, active lifestyles for patients and their families.

It is important to note that participants in this pilot study were recruited from the primary care clinic in the Center for CYSHCN at St. Christopher's Hospital for Children (SCHC), a large pediatric tertiary care hospital in an urban community. Therefore, the participants were medically stable even though many had significant health and environmental challenges. Some of the environmental challenges these families and CYSHCN face are due to the community in which they live (the neighborhoods surrounding and served by SCHC). SCHC is located in Eastern North Philadelphia, in Pennsylvania's 1st Congressional District. This area is described as having the third highest childhood poverty rate in the nation (45% compared to the national average of 22%), the second highest percentage of children living in single parent families in the nation (67% compared to the national average of 34%), the second most food insecure district in the nation (49.6% of households between 2008–2010), and the poorest neighborhood in the Commonwealth of Pennsylvania [30, 31].

The primary purposes of this pilot study were to describe child factors (weight status, diagnosis), child activity levels (physical activity and sedentary behaviors), and parent factors (parent education, income, and employment) and to identify social and environmental facilitators and barriers to physical activity for CYSHCN. A secondary purpose was to explore relationships among child and parent factors and child activity levels. We hypothesized that parent factors would be correlated with childhood weight status categories and activity levels. Further, we hypothesized that childhood weight status categories would be correlated with physical activity and sedentary behaviors. Lastly, we hypothesized that childhood weight status categories would be correlated with medical diagnoses or conditions [2, 18].

The final purpose of this study was to inform PCPs about community facilitators and barriers to physical activity for CYSHCN and their families so they can provide appropriate guidance and resources and advocate with and for families for more community resources to promote active, healthy lifestyles.

## 2. Materials and Methods

### 2.1. Participants

The participants in this study were CYSHCN (*n* = 23) and their parents or legal guardians (*n* = 21), including mothers (*n* = 17), one father, one foster father, and two grandmothers. Two parents each had two CYSHCN. Inclusion criteria were that children were ages 3–18 years, had a diagnosed special health care need(s), were medically stable and ambulatory, and were patients in the primary care clinic at the Center for CYSHCN at SCHC. Both parents and children needed to be proficient in English. A sample of convenience was recruited by the medical director (pediatrician) and nurse manager and participants were enrolled by the study team. Child and parent demographics can be found in Table 1.

Parent-child dyads were chosen to participate in this pilot study based on the evidence that parents are role models for their children, they provide opportunities for their children to participate in physical activities, and they may be more invested in their children's activities and services due to the demands of their child's health condition(s) [22, 23].

Information on children's diagnoses indicated that the most frequent primary diagnosis was autism (*n* = 7, 30%) followed by cerebral palsy (*n* = 3, 13%), and asthma (*n* = 2, 9%). Eleven children (48%) each had a unique primary diagnosis, and the majority were genetic syndromes and neuromuscular conditions. Moreover, most children had one or more diagnoses in addition to their primary diagnosis, which is listed on Table 2. Due to the heterogeneity of the children's primary diagnoses, the research team created two diagnostic categories; physical/medical conditions (*n* = 13, 57%) and cognitive/emotional/behavioral conditions (*n* = 10, 43%) (see Table 2).

Most parents (*n* = 14, 67%) indicated that their children used between 1 and 8 pieces of equipment or adaptive devices (mean = 1.3). Six children (33%) used nebulizers or portable inhalers and three children (13%) were on gastrostomy tubes. Of the three children with cerebral palsy, one was classified as Gross Motor Function Classification System level II (GMFCS II) and two were classified as GMFCS level III [32].

At the time of this study, 74% (*n* = 17) of children were on medications and parents reported that their children took up to seven prescribed medications (mean = 3.5). Six children (33%) used inhaled steroids, which may be associated with weight gain [33]. Five of these children were in the physical/medical group.

 Primary care providers (PCPs) often classify CYSHCN using the Complexity Index [34]. This tool uses a 10-point ordinal scale to rate the medical severity and social or family complexity of a child's condition [34]. CYSHCN in this study were assigned a rating by their PCP.  Table 3 shows the distribution of complexity scores for children in this study. We present these ratings to describe the participants and to provide information on the contextual factors (personal and social environment) contributing to the severity of their conditions. Note that all CYSHCN have moderate to severe medical problems and 35% (*n* = 8) also have complicating social or family issues.

### 2.2. Measures

**Figure 2:**
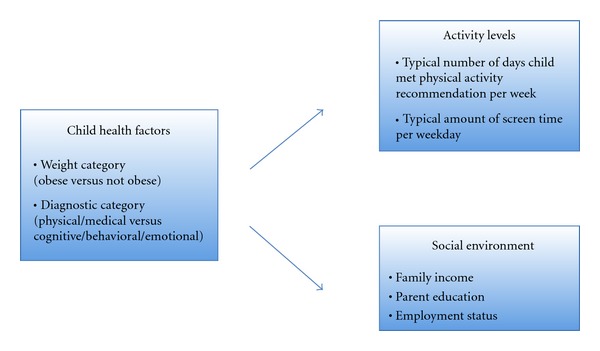
Items explored in correlation analysis.

This is a cross-sectional exploratory, descriptive study in which we examined the relationship among child, family, and activity variables (see Figure 2). Additionally we conducted in-depth interviews with families to gather qualitative data about community resources and barriers to physical activity for their CYSHCN. Each parent-child dyad participated in one data collection session, which included a series of questionnaires. Children were measured on height and weight to calculate body mass index (BMI).

**Child Medical Information Questionnaire (CMIQ)**

This questionnaire was used in past research on physical activity and fitness programs for CYSHCN [35, 36]. Parents completed this questionnaire to provide information on their children's diagnoses or medical conditions, medications, medical technology, seizures, allergies, or problems with vision, hearing, or communication. Other information obtained from this questionnaire included the child's past surgical interventions, exercise restrictions, use of assistive devices, home modifications, and child's participation in physical activity programs or on sports teams.

**Parent and Child Demographic and Health Behavior Questionnaires (PDHBQ and CDHBQ)**

These two questionnaires were developed for this study and were based on questionnaires used in past parent-child health behavior research on physical activity and sedentary behaviors [22]. The PDHBQ (36 items) and CDHBQ (31 items) used in the current study were revised to be more applicable to CYSHCN. Items on the PDHBQ included family structure, parent and child race and ethnicity, education and income levels, employment status, parent perceptions of child's overall weight and parent perceptions of their child's physical activity, and sedentary levels. Additionally, the PDHBQ had items for parents to rate availability and accessibility of physical activity resources in their neighborhood. The CDHBQ included items similar to the PDHBQ with additional items on perceptions of height and weight and ratings of overall health and desire to be healthier.

The health behavior items for both questionnaires were from the Youth Risk Behavior Surveillance Survey (YRBSS) for high-school and middle-school-aged students [37]. Some of the parent items were from the Behavioral Risk Factor Surveillance Survey (BRFSS) [38]. The specific items of interest for this study were the physical activity and sedentary behaviors items. We provided a definition of physical activity on the questionnaire to reduce ambiguity for the participants. The physical activity definition and PDHBQ items on physical activity and sedentary behavior are provided in Table 4.

Specific questions were asked of parents regarding what special assistance or adaptations children needed to be physically active. These adaptions may present as facilitators or barriers to a child's opportunities to participate in healthy activities. Using a 5-point rating scale, parents rated the degree to which an item presented as a barrier. Items included: child's mobility and health limitations, supervision and adaptive equipment in community programs, family finances to pay for adaptive equipment, and appropriate school-based physical education classes (see Table 5).

Lastly, to determine community resources and supports to promote health behaviors, parents were interviewed about availability and accessibility of physical activity resources in the families' community. Parents were also asked to identify health-promoting resources that they perceived to be absent from their community. Specific questions asked in the PDHBQ were as follows:

Please tell us what resources are available in your community to promote physical activity (e.g., *parks, fitness and recreation centers, summer camps, before or after school programs, public pools*.)
Are the above resources accessible? *(Meaning—Are they easy to get to? Are they open at convenient times? Are the prices affordable?) *
Are there any physical activity resources in community that you do not have, but would like to see?

**Body Mass Index (BMI)**

BMI was calculated from each child's anthropometric measures (height and weight). Children's height was measured using a stadiometer (Road Rod 214 Portable Stadiometer, SECA, Hanover, MD) and weight was measured with a digital scale (UMO 26; Tanita, Arlington Heights, IL). All children could maintain upright posture for height measures although several required two measurers, one to ensure the head and trunk were aligned and to position the head piece and take the measure, the other to support lower extremities with proper foot and knee alignment.

### 2.3. Procedure

#### 2.3.1. Recruitment and Enrollment

The PCP and nurse manager at SCHC approached families of children who met the inclusion criteria. Over the course of this one-year study, active recruitment and enrollment was conducted for eight months. A total of 45 parents gave permission to be contacted for participation in the study and 21 parents (47%) were enrolled. Approximately 250 phone calls were placed in an attempt to enroll the 45 parents. In many instances phone numbers were disconnected and parents were unable to be reached. Barriers for other parents who declined the invitation to participate were no time, difficulty accessing transportation, and lack of childcare. Forty-two appointments were scheduled to enroll the 21 participating parents. Reasons why scheduled appointments were missed included children being sick or hospitalized, parents forgetting, and poor weather conditions.

#### 2.3.2. Data Collection Session

Upon arrival to the clinic at SCHC, parents and CYSHCN were escorted to a private examination room in the clinic area. There was only one data collection session in the study. The session lasted approximately 90 minutes.

First, parents and children completed informed consent and assent forms, respectively. All questionnaires were administered by trained interviewers using the same sequence (CMIQ, PDHBQ, and CDHBQ). Most children required assistance from their parents to complete the CDHBQ. After completion of questionnaires, the child's anthropometric measures were taken. A standardized protocol was used to document height and weight and to calculate BMI [22].

Self-report measures were used in this study despite inherent biases in reporting because these are the usual measures to obtain demographic and descriptive information. Self-report measures are used most often to measures physical activity in CYSHCN with most evidence specific to children with cerebral palsy [39, 40]. Little evidence is available to support the validity or reliability of accelerometers or pedometers to measure physical activity in CYSHCN with available evidence specific to children and adolescent with cerebral palsy [41]. There are no studies that report using objective measures of sedentary behaviors for CYSHSN, so self-report is an appropriate measure [39, 40].

### 2.4. Data Analysis

A mixed methods design was implemented for descriptive and correlation analyses. The PASWStatistic 18.0 [42] statistical package was used to generate frequencies and correlations. Parents responses on the PDHBQ were used in the analysis. Child responses on the CDHBQ were not analyzed because of the amount of missing and incomplete data due to most children's inability to complete the CDHBQ (i.e., cognition and/or attention limitations). In situations where children are too young or lack the cognitive ability to respond on their own behalf, parents have been found to reliably report their child's health information [43].

Descriptive analyses were generated for child factors (weight status and diagnostic categories) and PDHBQ items (parent ratings of child's physical activity level and sedentary behavior; parent demographics (education, income and employment); parent ratings on facilitators and barriers to physical activity).

Chi-square analysis, Fisher's Exact Tests, and Spearman correlations were used to test the hypotheses and measure correlations among child factors (weight status, diagnostic category) parent factors (income, education, and employment); child activity level (physical and sedentary behaviors). The alpha level was set at *P* < 0.10 to reduce Type II error. Given the study design and sample size, the larger *P* value will allow us to see potential trends in correlations [44, 45].

Qualitative analysis was conducted to examine parent responses to the open-ended physical activity resource questions. These questions were administered via in-depth interviews and open-ended questions. Responses were coded based on themes and categories and frequencies were documented to identify unique trends or patterns [46]. Figure 2 illustrates the items examined in the association analysis. Only statistically significant findings are reported in the results section.

## 3. Results

### 3.1. Descriptive Analysis

#### 3.1.1. Body Composition, Physical Activity, and Child Health Factors

Many of the participants (*n* = 12, 55%) were obese (BMI ≥ 95th percentile). An additional 14% (*n* = 3) of participants were overweight (BMI ≥ 85th percentile). On average, parents reported that their children were physically active for 60 minutes or more on 4.68 (SD 2.0) days per week. None of the children in this study were reported to have participated on sports teams. Parents also reported that their children engaged in an average of 3.1 (SD 2.3) hours of screen time per day. Screen time was defined as television, video games, and computer use (for nonhomework activities) [47].

#### 3.1.2. Parent Perception of Child's Overall Health

Parents used a 5-point Likert scale to rate their child's overall health and to rate how much their child's health or physical limitations were a barrier to physical activity. A majority (68%, *n* = 15) of parents rated their children in good, very good, or excellent overall health. A majority of parents (55%, *n* = 12) indicated that mobility limitations were rarely or never a barrier.

#### 3.1.3. Environmental Facilitators and Barriers to Physical Activity

Parents were asked to rate the level by which specific social, organizational, and community resources or lack of resources presented as barriers to physical activity. Parents were asked to rate each item (presented in Table 5) on a 5-point Likert scale from “never” to “always” a barrier. A majority of parents perceived the level of supervision in community programs as a barrier. Additionally, a majority of parents agreed that limited finances to pay for adaptive equipment were also a barrier.

Parents were asked to identify available and accessible resources in their community to support physical activity behaviors for their children. Availability was defined as resources that exist in the community and accessibility was defined as, affordable, convenient hours of operation, easy to get to, and adaptable to the specific needs of their child. Parents were also asked what community resources were needed to support physical activity. When examining open-ended responses to these questions, parks were the most frequently identified physical activity resource in parents' communities. In addition, most parents perceived these parks to be an accessible resource to promote physical activity for their CYSHCN. On the other hand, pools are needed in many parents' communities. Twenty-one percent of parents said that they do not have a pool in their community, but would like one. Pools only accounted for five percent of the available resources cited by parents. A complete summary of responses is displayed in Table 6.

### 3.2. Factors Associated with Obesity in CYSHCN

Results of the chi-square analyses (Table 7) suggest that CYSHCN who were classified as having cognitive/behavioral/emotional conditions were more likely to be obese compared to CYSHCN who were classified as having physical/medical conditions (*P* = 0.10). CYSHCN who belong to families of higher income were more likely to be obese compared to CYSHCN who belonged to families of lower income (*P* = 0.06).

Results of the Spearman's correlation suggest that BMI percentile was significantly, inversely related to the number of days in which a child achieves recommended levels of physical activity in a typical week (*r* = −.43, *P* < 0.05). 

## 4. Discussion

A majority of CYSHCN in this study were obese. This finding is supported by previous studies demonstrating a high prevalence of overweight and obesity in CYSHCN [2, 18]. We hypothesized that parent factors would be correlated with child weight status category and activity levels. Additionally, we hypothesized that child weight status would be correlated with activity levels and diagnostic category.

There were three significant findings from the hypothesis testing. Children who were categorized with cognitive/emotional/behavioral conditions were significantly more likely to be obese than those categorized with physical/medical conditions. This finding is supported by previous studies of CYSHCN [18] and may highlight the need for more accessible facilities, family and recreation or fitness staff training, and adapted activities to provide safe, appropriate environments with sustained moderate-to-vigorous physical activity to result in improved health and weight status. Further research is necessary to understand health outcomes and long-term implications in this specific population of CYSHCN.

The second significant finding from hypothesis testing suggests that there was a significant relationship between increased parent income and increased obesity. This finding is counterintuitive but may be partially explained by the fact that most of the CYSHCN in the higher-income families had cognitive/emotional/behavioral conditions and children with these conditions were significantly more obese than children with physical/medical conditions.

Finally, the significant inverse correlation between obesity and physical activity is in keeping with the research evidence and suggests that inactivity in CYSHCN may lead to obesity [18]. On average, parents indicated that their children did not achieve the recommended level of daily moderate-to-vigorous physical activity (60 minutes every day) [48]. Data for typical high school students suggests that only 18.4% achieve the recommended daily levels of moderate-to-vigorous physical activity [49] but this is much better than our findings of 0%. Although CYSHCN did not meet the physical activity recommended threshold, parents responses were higher than expected and were not supported by the high obesity rates in the participants. A possible explanation is that the physical activity parents observed may not be at the necessary intensity (moderate to vigorous) to result in energy expenditure for CYSHCN to achieve and maintain healthy weight. In candid conversations during interview sessions, some parents spoke of their children not being able to fully participate in sports and recreation to the degree and competitive level of children with typical development. Also, as mentioned previously, we must consider the biases inherent in self-report data [50].

All CYSHCN in this study engage in screen time beyond the American Academy of Pediatrics recommendation (1-2 hours per day) [50]. In our study, CYSHCN participated in less screen time compared to typically developing children. Evidence suggests that preschoolers with typical development are exposed up to 4 hours of screen time per weekday, and older children and adolescents are exposed up to approximately 3 hours of television per day, not including time spent with videogames and computers [51–53]. Other studies have found that children with cognitive disorders, such as autism, spend a greater amount of their free time watching television or playing video games than their peers with typical development [54]. Children with cerebral palsy have been found to spend relatively the same amount of time engaged in screen time as their peers with typical development [55]. Findings from our study suggest that CYSHCN participate in less screen time than children with typical development and less screen time than reported in other studies with CYSHCN. This discrepancy may be due to parent report or that the item on screen time in the PDHBQ potentially caused confusion. We asked parents to add up their children's screen time, which may have lead to inaccuracy in adding those numbers. There are limited studies investigating screen time in CYSHCN, this health behavior warrants future research.

In this study, parents identified multiple barriers to physical activity, with a lack of supervision in community programs being the most common concern. A majority of parents agreed that limited finances and inability to pay for adaptive equipment posed barriers to their children being able to participate in physical activities. Additionally parents identified multiple resources they needed in their community to promote physically activity for their children, including pools, gyms, and summer camps.

It is worth noting that no parents in this study had a playground in their community that was accessible to their CYSHCN. Since the average age of children in our study was 9.8 years and many children had cognitive delay, it is likely that by cognitive or developmental age, playgrounds may be useful to these families. Lack of playground resources speaks to potential problems with resource availability and/or appropriate adaptations for universal access for all children. National physical activity guidelines [48] should include recommendations on ways to adapt environments to promote active recreation in all children to make the guidelines more comprehensive and inclusive. Although these guidelines mention adults and CYSHCN, minimal recommendations are provided to address the health and physical activity needs of these populations [48]. Special attention and detail in national guidelines and clinical guidelines may help guide PCPs in their health promotion recommendations to CYSHCN. Likewise, citywide health promotion initiatives should include information and recommendations on universal access to include CYSHCN and their families so they can participate in and benefit from these initiatives as much as children with typical development and their families can.

## 5. Limitations

A main limitation in this study is the small sample size that resulted from difficulty with recruitment and enrollment. Data analysis was therefore limited to descriptive statistics and nonparametric correlation analyses. Subjects enrolled in the study were a heterogeneous population of CYSHCN making it impossible to examine specific conditions or diagnoses as related to weight status or physical activity levels. Likewise, the socioeconomic status of participant families varied but the majority of families were from the lower end of the socioeconomic status making it impossible to generalize across all income levels of families with CYSHCN.

As in many studies, there is the potential for selection bias, and the parent and child health questionnaires brought inherent self-report bias. Lastly, the 36-item PDBHQ and 31-item CDBHQ may have been too long and burdensome. Upon observation it appeared that parents and children might not have answered questions with the same interest level near the end of the interview. Through the course of data collection, it was determined that the CDBHQ was not appropriate for most CYSHCN in this study. If this study were to continue, only the PDBHQ would be used. A major revision would be necessary for the CDBHQ if it were to be used again.

## 6. Conclusion

To our knowledge, this is the first study conducted in primary care to examine parent perspectives and community resource needs to promote physical activity in CYSHCN. Also, this clinic-based study was conducted with a vulnerable population living in an underserved community. Additionally, this study contributes to the limited literature documenting overweight and obesity CYSHCN. Parents identified multiple resources in their communities to promote physical activity. PCPs should provide families with anticipatory guidance and recommendations to find those resources for health promotion and participation in healthy, active recreation. PCPs for CYSHCN who use the Complexity Index Scale to describe their patients should consider adding functional items to the scale to identify physical activity limitations and needs for their patients and families to promote healthy weight and active lifestyles. Physical and occupational therapists working in primary care clinics for CYSHCN should be included as part of the medical team to provide these children and families with resources, equipment ideas, and program ideas to promote healthy participation in physical activity and active recreation. Resources that were reported as a “need” in communities should be brought to the attention of local policy makers, PCPs, and advocates of CYSHCN. It is important that parents recognize the prevalence of overweight and obesity in CYSHCN and assist their children to engage in physical activity for health promotion and prevention of secondary conditions. Likewise, it is important that universally accessible community resources are available to CYSHCN to promote health behaviors.

## Figures and Tables

**Table 1:** Parent/guardian and child demographics.

Variable	Child	Parent
*Gender n* (%) (Child *n* = 23, Parent, *n* = 21)		
Male	17 (74)	2 (10)
Female	6 (26)	19 (90)

*Age* mean (sd, range) (Child, *n* = 23, Parent, *n* = 20)	9.7 (4.64, 3–18)	38 (16.17, 24–58)

*Race n* (%) (Child, *n* = 23, Parent, *n* = 20)		
Asian	1 (4)	1 (5)
Black/African American	6 (26)	5 (25)
Native Hawaiian/Pacific Islander	1 (4)	1 (5)
White	5 (22)	5 (25)
Other	10 (43)	8 (40)

*Annual household income *(*n* = 17) *n* (%)		
<$15,000	NA	7 (41)
$15,000–29,999	NA	4 (24)
$30,000–$44,999	NA	4 (24)
$45,000–59,999	NA	1 (6)
$75,000–$99,999	NA	1 (6)

*Child health factors* mean (sd)		
Average BMI percentile (*n* = 22)	80 (31)	NA
Average number of days child achieves recommended level of physical activity (60 minutes of moderate-to-vigorous physical activity) (*n* = 22)	4.7 (1.9)	NA
“Screen time” (average no. of hours/typical weekday) (*n* = 21)	3.1 (2.3)	NA

**Table 2:** Primary diagnoses and diagnostic categories.

Primary diagnosis	Number of children	Diagnosis category
Autism	7	Cognitive/emotional/behavioral
Cerebral palsy	3	Physical/medical
Asthma	2	Physical/medical
ADHD	1	Cognitive/emotional/behavioral
Mood disorder	1	Cognitive/emotional/behavioral
Severe intellectual disability	1	Cognitive/emotional/behavioral
Charge syndrome	1	Physical/medical
Premature	1	Physical/medical
Beckwith-Wiedemann syndrome	1	Physical/medical
Noonan syndrome	1	Physical/medical
Spina bifida	1	Physical/medical
Seizures	1	Physical/medical
Brain tumor	1	Physical/medical
Spherocytosis	1	Physical/medical

**Table 3:** Complexity Index Scores.

Complexity	Description	*n* (%)
0	Well child	0
0S	Well, no medical problems, but does have complicating social or family issues	0
1	One moderate medical problem involving one organ system	0
1S	One moderate medical problem involving one organ system with complicating social or family issues	0
2	One moderate or severe medical problem, involving one organ system with complications	5 (21.7)
2S	One moderate or severe medical problem, involving one organ system with complications and with complicating social or family issues	1 (4)
3	Two or more moderate or severe medical problems, involving two or more organ systems	9 (40)
3S	Two or more moderate or severe medical problems, involving two or more organ systems and with complicating social or family issues	5 (21.7)
4	Two or more moderate or severe medical problems, involving two or more organ systems with complications	1 (4)
4S	Two or more moderate or severe medical problems, involving two or more organ systems with complications and with complicating social or family issues	2 (8.6)

“S” indicates a child with a psychosocial issue or factor in the household (includes but not limited to single parent, foster care, and history of domestic violence) [27].

**Table 4:** PDHBQ items used in analysis.

Definitions	Items
(i) *Physical activity* is any activity that increases your child's heart rate and makes him or her get out of breath some of the time. (ii) *Physical activity* can be done in sports, playing with friends, or walking to school. (iii) Some examples of *physical activity* are running, brisk walking, rollerblading, biking, dancing, skateboarding, swimming, playing soccer, basketball, or football, and surfing.	Over a *typical or usual week*, on how many days is *your child * *physically active* for a total of at least 6*0 minutes* per day? 0 day 1 day 2 days 3 days 4 days 5 days 6 days 7 days

Sedentary behavior was defined as time spent in “screen time.” Screen time includes time spent watching the television, playing video games, and using the computer for nonhomework reasons.	How many hours is your child engaged in screen time on a school day? (a) My child does not watch TV, play video games, or use the computer on school days (b) Less than 1 hour per day (please specify number of minutes):—— (c) 1 hour per day (d) 2 hours per day (e) 3 hours per day (f) 4 hours per day (g) 5 hours per day

**Table 5:** Most common barriers to physical activity.

Item	Sometimes to always a barrier *n* (%)
(a) My child needs more supervision than is usually available in community programs (*n* = 22)	16 (73)
(b) My limited finances to pay for adaptive equipment for my child (*n* = 20)	12 (60)
(c) The lack of school-based physical education and/or activity programs that are appropriate for my child to participate in physical activity (*n* = 19)	9 (47)
(d) My child's mobility limitations or fragile health (*n* = 22)	10 (46)
(e) The lack of adaptive equipment in community programs to help my child participate (*n* = 21)	5 (24)

**Table 6:** Availability, accessibility and need of physical activity resources in the community.

Resource	Available	Accessible	Need
Parks	35%	80%	0%
*Other	18%	90%	NA
Recreation Centers	16%	90%	7%
Gyms	11%	67%	10%
YMCA	9%	100%	14%
Pools	5%	100%	21%
Playgrounds	5%	0%	10%
Sports	0%	0%	7%
Summer Camps	0%	0%	7%
**Needs	NA	NA	24%

*Other refers to available/accessible resources (such as church, bowling, variety club) that were only mentioned once.

**Needs refers to needed resources (such as horse backing riding, gymnastics and after school programs) that were only mentioned once.

**Table 7:** Chi-square analysis.

**Diagnostic category**(*n* = 22)	Obese *n* (%)	Not obese *n* (%)	Exact sig (2-sided)*
Physical/medical	5 (42)	7 (58)	*P* = 0.10
Cognitive/behavioral/emotional	8 (80)	2 (20)	

**Family income category **(*n* = 17)	Obese *n* (%)	Not Obese *n* (%)	Exact Sig (2-sided)*

<$15,000 per year	2 (29)	5 (71)	*P* = 0.06
≥$15,000 per year	8 (80)	2 (20)	

*Fisher's Exact Test.
